# Machine Learning-Based Prediction of Surgical Intervention in Preterm Infants with Necrotizing Enterocolitis: A Retrospective Cohort Study

**DOI:** 10.3390/children13010021

**Published:** 2025-12-22

**Authors:** Ying Li, Peipei Zhang, Jing Wu, Ying Wang, Ying Chen, Sihan Sheng, Yajuan Wang, Xiaohui Li

**Affiliations:** 1 Capital Institute of Pediatrics, Chinese Academy of Medical Sciences & Peking Union Medical College, Beijing 100730, China; xsely@mail.ccmu.edu.cn (Y.L.);; 2 Department of Neonatal Intensive Care Unit, Capital Center for Children’s Health, Capital Medical University, Beijing 100020, China; 3 Center for Evidence-Based Medicine, Capital Center for Children’s Health, Capital Medical University, Capital Institute of Pediatrics, Beijing 100020, China; 4 Department of Neonatology Surgery, Capital Center for Children’s Health, Capital Medical University, Beijing 100020, China

**Keywords:** necrotizing enterocolitis, preterm infants, surgical intervention, machine learning, SHAP interpretability

## Abstract

**Background:** Necrotizing enterocolitis (NEC) is a life-threatening gastrointestinal disorder in neonates, particularly preterm infants. Early identification of infants requiring surgical intervention remains challenging due to nonspecific clinical manifestations and rapid disease progression. **Methods:** We conducted a retrospective cohort study of 320 preterm infants with NEC (gestational age <37 weeks) who were admitted to the NICU of the Capital Center for Children’s Health, Capital Medical University, Beijing, China, between June 2017 and December 2024. Forty-three clinical, laboratory, and imaging variables were collected. Feature selection was performed using LASSO regression and the Boruta algorithm. Four machine learning (ML) models—LightGBM, XGBoost, Random Forest, and Neural Network—were constructed. Model performance was evaluated using ROC-AUC, PR-AUC, accuracy, sensitivity, specificity, positive predictive value (PPV), negative predictive value (NPV), and SHAP-based interpretability. **Results:** Among 320 infants, 119 underwent surgery and 201 received non-operative management. Thirteen consensus features were selected for modeling, including gestational age, CRP, lactic acid, peritoneal irritation signs, pneumatosis intestinalis, and hematologic parameters. The Neural Network achieved the highest overall classification performance (accuracy 0.875, sensitivity 0.824, specificity 0.903, balanced accuracy 0.863); Random Forest achieved the highest ROC-AUC (0.922), and XGBoost showed the highest PR-AUC (0.867). SHAP analysis identified CRP, peritoneal irritation signs, and gestational age as the most influential predictors. **Conclusions:** ML models integrating clinical, laboratory, and imaging variables can accurately predict the need for surgical intervention in preterm NEC patients. These models provide objective decision-support tools to improve early identification and optimize surgical management.

## 1. Introduction

Necrotizing enterocolitis (NEC) is a potentially life-threatening gastrointestinal disorder that predominantly affects neonates with a particularly high incidence among preterm infants, especially those born before 32 weeks of gestation [1]. While approximately 10% of NEC cases occur in full-term infants, these patients often present with comorbidities such as congenital heart disease, gastrointestinal anomalies, chromosomal abnormalities, or sepsis [2]. Preterm and term infants differ substantially in clinical manifestations and disease progression, with term infants exhibiting lower rates surgical intervention, highlighting the need for tailored management strategies [3,4].

International studies have reported variability in the incidence of Bell stage II or higher NEC among very preterm infants, ranging from 1.7% in Japan to 6.9% in Spain [5,6]. Approximately one-third of infants weighing <1500 g or born before 32 weeks require exploratory surgery. Among surgically managed cases, mortality may reach 50%, and survivors frequently develop short- or long-term complications, including growth failure, short bowel syndrome, and neurodevelopmental impairments [7].

Clinical management of NEC is challenging because of its nonspecific early symptoms and rapid progression. Surgical indications are currently determined based on Bell staging, imaging findings, and laboratory parameters. The timing and appropriateness of surgery critically influence prognosis, yet decisions rely largely on clinical judgment, and lack objective predictive tools.

Previous studies have explored clinical, biomarker, and imaging predictors of NEC severity, but individual indicators show limited sensitivity and specificity. Multivariable models, particularly conventional logistic regression, fail to capture nonlinear relationships, suffer from heterogeneity, and demonstrate suboptimal clinical applicability [8,9,10,11].

Thus, there is an unmet need for robust predictive models to assist in NEC surgical decision-making. Machine learning (ML) has the potential to meet this need, but its application to NEC remains limited. To address this gap, we integrated comprehensive clinical, laboratory, and imaging data to construct ML-based models for predicting surgical intervention in preterm patients with NEC.

## 2. Methods

### 2.1. Study Design and Participants

This retrospective cohort study enrolled premature infants diagnosed with NEC in the Neonatal Intensive Care Unit (NICU) of the Capital Center for Children’s Health, Capital Medical University, Beijing, China, between June 2017 and December 2024. NEC diagnosis was confirmed according to modified Bell’s staging criteria (Stage ≥ II) and aligned with the 5th edition of Practice of Neonatology [12] and relevant expert consensus [13]. Patients were stratified into operative and non-operative management groups. The study was approved by the Institutional Ethics Committee (Approval No.: SHERLLM2024043; Approval Date: [29 October 2025]), with informed consent waived due to the retrospective nature of the study and the use of de-identified data.

Inclusion Criteria: (1) Neonates diagnosed with NEC meeting modified Bell’s staging criteria ≥Stage II; (2) premature infants with a gestational age < 37 weeks (259 days); (3) availability of complete and verifiable medical records, including clinical, laboratory, and imaging data; (4) hospitalization duration > 24 h following the initial diagnosis.

Exclusion Criteria: (1) Hospital admission duration < 24 h for any reason; (2) incomplete or inaccessible medical documentation; (3) comorbid major congenital anomalies, significant intestinal malformations (e.g., Hirschsprung’s disease, intestinal atresia, malrotation), or spontaneous intestinal perforation; (4) patients for whom active medical care was withdrawn or who died prior to enrollment.

Surgical intervention for NEC was defined as any laparotomy and/or bowel resection performed for the disease. At our institution, the main indications for surgery were (1) confirmed or strongly suspected intestinal perforation, such as pneumoperitoneum or free intraperitoneal air on abdominal radiography, and/or (2) the presence of yellow-brown or bile-stained ascitic fluid obtained by diagnostic abdominocentesis, suggesting bowel necrosis or perforation. The final decision to proceed to surgery was made by the attending pediatric surgeon in consultation with the neonatology team.

### 2.2. Data Collection and Preprocessing

A standardized data collection form was used to extract information from electronic medical records. Feature selection was guided by established clinical expertise and systematic evidence from the NEC operative decision-making literature, culminating in 43 variables for the final analysis. All clinical, laboratory, and imaging variables were obtained at the time of NEC diagnosis (Bell stage ≥ II) and before any surgical intervention or operative decision-making. These variables encompassed the following domains: (1) demographics and perinatal factors: gender, age, gestational age at birth, birth weight, intrauterine distress, premature rupture of membranes (PROM), amniotic fluid characteristics, placental abnormalities, feeding type; (2) pharmacological exposures: pulmonary surfactant, caffeine, glucocorticoids, vasoactive drugs, diuretics, intravenous immunoglobulin (IVIG), oral probiotics, antibiotics; (3) comorbidities and clinical interventions: infectious diseases, patent ductus arteriosus (PDA), mechanical ventilation, central venous catheterization; (4) clinical manifestations: NEC onset time, decreased bowel sounds, gastric retention, abdominal distension, tachycardia, hematochezia, peritoneal irritation, abdominal wall erythema, abdominal mass, hypotension; (5) laboratory and imaging parameters: metabolic acidosis, lactic acid, C-reactive protein (CRP), neutrophil percentage, hemoglobin, platelet count, hyponatremia, positive blood culture, pneumatosis intestinalis, portal venous gas, intestinal wall thickening, intestinal dilation. All abdominal radiographs and ultrasound examinations were interpreted by attending pediatric radiologists who were blinded to whether the infants subsequently underwent surgical intervention and were not informed of the study hypothesis.

### 2.3. Feature Selection for Machine Learning Analysis

To ensure robust model performance, missing values were handled using multivariate imputation by chained equations (MICE) implemented in the “mice” package in R. Under the missing-at-random assumption, five imputed datasets (*m* = 5) were generated using the MICE default settings, which apply predictive mean matching for continuous variables and logistic or polytomous regression models for binary and multicategorical variables, respectively. All candidate predictors together with the outcome variable (surgical intervention) were included in the imputation model. Continuous features were subsequently standardized using the Z-score method to maintain a consistent scale across variables, facilitating fair comparisons in subsequent modeling. For feature selection, we intentionally combined two complementary approaches to capture both linear and potentially nonlinear relationships with the outcome and to enhance robustness. First, we applied LASSO (Least Absolute Shrinkage and Selection Operator) logistic regression, which shrinks regression coefficients via an L1 penalty [14], thereby performing variable selection while mitigating multicollinearity and yielding a parsimonious set of predictors. Second, we used the Boruta algorithm, a Random Forest–based wrapper method that evaluates variable importance in a nonparametric manner and can model complex interactions and nonlinear effects [15]. The final feature set was defined as the intersection of variables selected by both methods, representing predictors consistently identified as important across distinct modeling paradigms. For each train–test split and cross-validation resample, multiple imputation, standardization, and feature selection were carried out using only the training data; the derived imputation models and scaling parameters were then applied to the corresponding validation or test sets, which were not used for model tuning or feature selection. Thus, all machine learning models were fitted and evaluated on fully imputed and preprocessed data while avoiding information leakage from the test set.

In addition, to evaluate the robustness of the selected feature set, we performed a bootstrap-based feature-selection stability analysis: using the full NEC cohort (*n* = 320), we generated 200 bootstrap resamples, refitted a LASSO logistic regression model in each resample, and recorded whether each of the 13 predictors was re-selected (non-zero coefficients) to obtain selection frequencies.

### 2.4. Machine Learning Model Construction and Evaluation

Four machine learning models were constructed: the Light Gradient Boosting Machine (LightGBM), eXtreme Gradient Boosting (XGBoost), Random Forest, and Neural Network. The final modeling dataset contained continuous variables and binary clinical indicators coded as 0 or 1 (e.g., presence or absence of abdominal distension, tachycardia, and pneumatosis intestinalis); therefore, categorical predictors were already represented as dummy variables, and no additional one-hot encoding step was required. The dataset was split into training and testing sets at a 7:3 ratio. This split was performed before any imputation, scaling, or feature selection, and all such preprocessing steps were conducted with the training data (and within each training fold during cross-validation) and then applied to the held-out test set. Model training included hyperparameter optimization, early stopping (where applicable), and 5-fold cross-validation. Model performance was evaluated using the receiver operating characteristic curve (ROC) with area under the curve (AUC), accuracy, sensitivity, specificity, precision, positive predictive value (PPV), and negative predictive value (NPV). To quantify uncertainty in model discrimination and enable formal comparison between algorithms, we performed nonparametric bootstrap resampling of the test set (200 replicates) for each ML model and obtained 95% confidence intervals for ROC-AUC and PR-AUC. Pairwise differences in ROC-AUC between the four models were examined using DeLong’s test with *p*-values. Given the borderline events-per-variable ratio in the smaller (non-surgical) class, we further examined the robustness of our primary LightGBM model using learning curves and bootstrap resampling. For the learning curves, the training size was sequentially increased from 20% to 40%, 60%, 80%, and 100% of the training set (approximately 44–224 patients), with 30 random subsamples at each size; in every subsample, a LightGBM model with tuned hyperparameters was trained and evaluated on the same held-out test set, and mean ROC-AUC and PR-AUC were plotted against training size. For uncertainty quantification, 95% confidence intervals for ROC-AUC and PR-AUC of the LightGBM model were obtained by nonparametric bootstrap resampling of the test set (200 bootstrap samples with replacement), recomputing ROC-AUC and PR-AUC in each replicate using the precrec framework.

Finally, to evaluate clinical utility, we the performed decision-curve analysis (DCA) for all four ML models over threshold probabilities from 0 to 0.50 using the dcurves package, and for LightGBM, we extracted the net benefit and threshold-specific operating characteristics at probability cutoffs of 0.30 and 0.50.

### 2.5. Statistical Analyses

The baseline characteristics of the study participants by DDs were compared using one-way ANOVA, Kruskal–Wallis, and χ^2^ tests, where appropriate. Continuous variables are presented as medians (Q_1_, Q_3_) and categorical variables as numbers (percentages). Continuous variables are expressed as median (Q_1_, Q_3_) and were compared using the Mann–Whitney U test. Categorical variables are presented as frequencies (percentages) and were analyzed using the χ^2^ test. Statistical significance was defined as *p* < 0.05 (two-tailed). Feature selection and ML analyses were performed using R (v4.3.3) with the “mlr3proba” package for survival modeling. Model interpretability was visualized with SHAP frameworks.

## 3. Results

### 3.1. Characteristics of Study Participants

**Figure 1:**
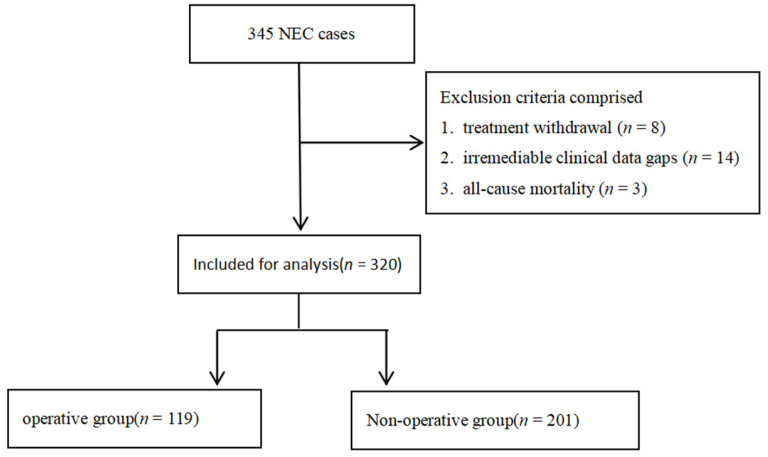
Flowchart of study population selection.

This study enrolled 345 infants according to inclusion and exclusion criteria from the Department of Neonatal Medicine, Pediatric Surgery, and the Intensive Care Unit at our institution between June 2017 and December 2024. After excluding 8 cases due to treatment withdrawal, 14 patients with incomplete clinical documentation, and 3 mortalities, 320 patients were ultimately included in the analysis. The cohort comprised 119 operative intervention cases and 201 non-operative management cases. The patient enrollment pathway is detailed in Figure 1.

The final analysis included 320 neonates with NEC, of whom 119 received operative management and 201 were managed non-operatively (Table 1, Table 2, Table 3 and Table 4). Compared with non-operative patients, neonates in the operative group had a lower age (median 7.0 vs. 10.0 days, *p* = 0.02) but higher gestational age (239.0 vs. 225.0 days, *p* < 0.001) and birth weight (2040 vs. 1600 g, *p* < 0.001). Feeding type differed significantly between groups (*p* = 0.04), with mixed feeding being more common in the operative group.

Clinically, operative patients exhibited higher rates of abdominal distension (94.96% vs. 49.25%, *p* < 0.001), decreased bowel sounds (85.71% vs. 57.21%, *p* < 0.001), peritoneal irritation signs (46.22% vs. 5.47%, *p* < 0.001), and abdominal wall erythema (13.45% vs. 0.50%, *p* < 0.001). Conversely, tachycardia (26.05% vs. 5.97%, *p* < 0.001) and hypotension (15.97% vs. 2.50%, *p* < 0.001) were more frequent in non-operative patients. Hematochezia was also more prevalent in the operative group (49.25% vs. 36.13%, *p* = 0.02).

Laboratory and imaging findings further distinguished the groups. Operative patients had markedly higher CRP levels (43.0 vs. 5.0 mg/L, *p* < 0.001) and a lower neutrophil percentage (40.4% vs. 57.6%, *p* < 0.001), while platelet count (279.0 vs. 172.0 × 10^9^/L, *p* < 0.001) and hemoglobin (135.0 vs. 118.0 g/L, *p* < 0.001) were elevated. Metabolic acidosis (27.12% vs. 6.60%, *p* < 0.001), hyponatremia (41.18% vs. 22.22%, *p* < 0.001), pneumatosis intestinalis (79.60% vs. 55.46%, *p* < 0.001), and portal venous gas (33.33% vs. 19.33%, *p* = 0.007) were significantly associated with operative management.

### 3.2. Feature Selection for ML

**Figure 2:**
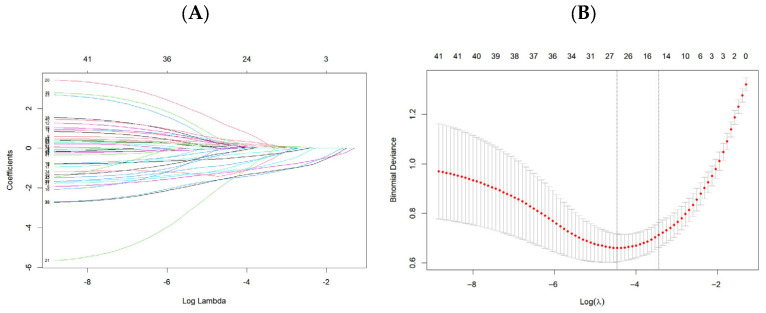

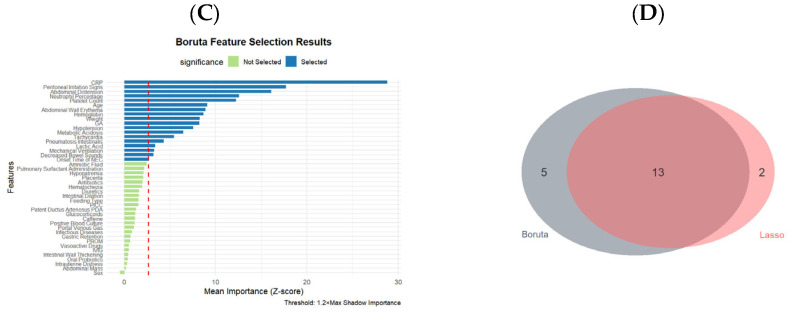
Feature selection using LASSO regression and Boruta. (**A**) LASSO coefficient profiles for 43 candidate predictors as a function of the log-transformed penalty parameter λ (*x*-axis). The *y*-axis represents the estimated regression coefficients. (**B**) Ten-fold cross-validation curve for LASSO, showing the mean binomial deviance (*y*-axis) across different values of log(λ) (*x*-axis); the vertical dashed line indicates the optimal λ. (**C**) Boruta feature importance plot, where the *x*-axis shows the Z-score of importance and the *y*-axis lists the clinical variables. Blue boxplots represent confirmed important features, and green boxplots indicate rejected features. (**D**) Venn diagram illustrating the overlap and method-specific features selected by LASSO and Boruta.

A total of 43 clinical variables potentially influencing surgical decision-making in NEC were collected based on clinical experience and previous studies. LASSO regression selected 15 variables, while the Boruta algorithm identified 18 variables. Among these, 13 predictors were shared by both methods, whereas 2 and 5 variables were unique to LASSO and Boruta, respectively. From the intersection of the two approaches, 13 consensus features were ultimately selected: gestational age (GA), lactic acid, C-reactive protein (CRP), neutrophil percentage, hemoglobin, platelet count, abdominal distention, tachycardia, peritoneal irritation signs, abdominal wall erythema, hypotension, metabolic acidosis, and pneumatosis intestinalis (Figure 2D). These selected predictors were subsequently used to develop four machine learning classifiers: Light Gradient Boosting Machine (LightGBM), eXtreme Gradient Boosting (XGBoost), Random Forest, and Neural Network.

### 3.3. Evaluation of ML Models

**Figure 3:**
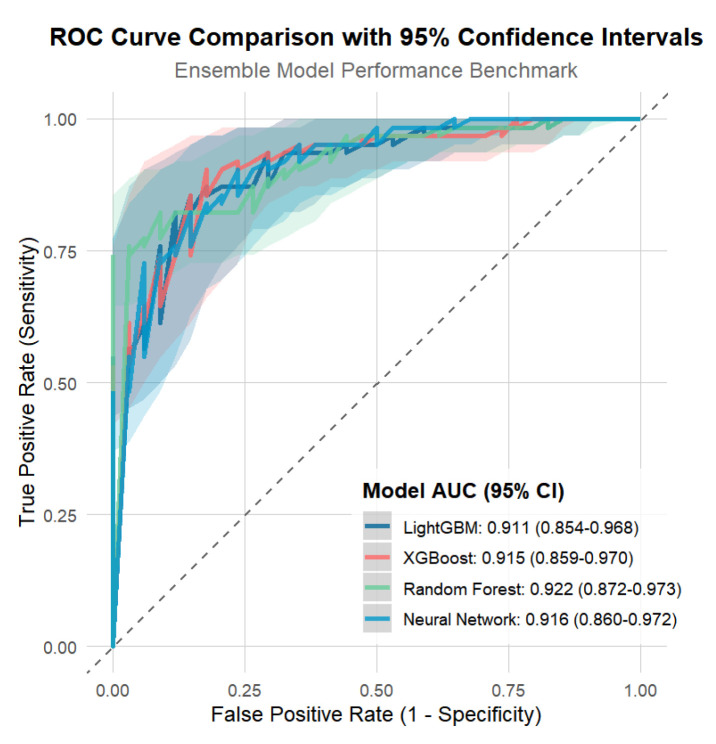
The ROCs of different machine learning models for predicting surgical necrotizing enterocolitis. AUC, area under the curve; LightGBM, Light Gradient Boosting Machine; RF, Random Forest; XGBoost, eXtreme Gradient Boosting Machine.

The performance of the four machine learning models—LightGBM, XGBoost, Random Forest, and Neural Network—is summarized in Figure 3 and Table 5. The Neural Network demonstrated superior performance across most classification metrics, achieving the highest accuracy (0.875), sensitivity (0.824), specificity (0.903), positive predictive value (PPV, 0.824), negative predictive value (NPV, 0.903), and balanced accuracy (0.863).

Regarding discriminative ability, Random Forest exhibited the highest area under the receiver operating characteristic curve (ROC-AUC, 0.922), followed closely by the Neural Network (0.916), XGBoost (0.915), and LightGBM (0.911). Bootstrap 95% confidence intervals for ROC-AUC were 0.851–0.961 for LightGBM, 0.852–0.962 for XGBoost, 0.870–0.967 for Random Forest, and 0.861–0.967 for the Neural Network. For the precision–recall curve, XGBoost achieved the highest PR-AUC (0.867), with the Neural Network, Random Forest, and LightGBM showing slightly lower values (0.851, 0.847, and 0.843, respectively).

To further assess robustness in the context of a borderline events-per-variable ratio, we examined learning curves and bootstrap confidence intervals for the LightGBM model. As shown in Supplementary Figure S1 ROC-AUC increased from approximately 0.50 at 20% of the training data to 0.91 at 100%, while Figure S2 PR-AUC increased from 0.35 to 0.84; beyond 60% to 80% of the training size, both metrics plateaued, with only marginal further improvements, indicating that the current sample size is sufficient for the model to learn a stable decision boundary. Nonparametric bootstrap resampling of the test set (200 replicates) yielded relatively narrow 95% confidence intervals for LightGBM performance. In addition, the bootstrap-based feature selection stability analysis showed that all 13 predictors were re-selected by LASSO in at least 92.5% of 200 bootstrap resamples (range: 92.5–100%), with lactic acid, C-reactive protein, neutrophil percentage, abdominal distension, peritoneal irritation signs, and metabolic acidosis being selected in 100% of resamples, supporting the stability of the final feature set. DeLong’s test for pairwise differences in ROC-AUC between the four models did not identify any statistically significant contrasts (all *p* ≥ 0.55), suggesting that the apparent differences in AUC are largely attributable to sampling variability rather than true performance gaps.

Overall, while the Neural Network outperformed the other models in most threshold-dependent classification metrics, differences in ROC-AUC and PR-AUC among the four algorithms were modest and not statistically significant. We therefore selected LightGBM as the primary reference model for learning-curve, feature-stability, and clinical-utility analyses because of its competitive discrimination, stable calibration, and favorable interpretability.

**Figure 4:**
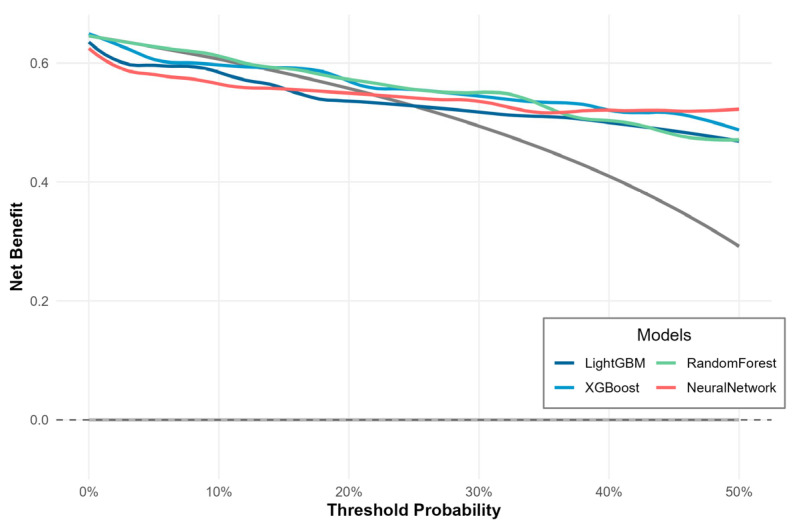
Decision-curve analysis for the four machine learning models.

Decision-curve analysis further demonstrated the potential clinical utility of the models (Figure 4). Across the clinically relevant threshold probability range from 0 to 0.50, the net-benefit curves of all four ML models lie above those of both a “treat-all” strategy (assuming that all infants require surgery) and a “treat-none” strategy, with LightGBM and the Neural Network showing almost overlapping and slightly higher net benefits than XGBoost and Random Forest. For LightGBM, two representative decision thresholds were examined in more detail. At a threshold of 0.30 for the predicted probability of surgical NEC, 66.7% (64/96) of infants in the test set would be classified as high-risk; the corresponding sensitivity, specificity, PPV, and NPV were 0.871, 0.706, 0.844, and 0.750, respectively, with a net benefit of 0.518 compared with 0.494 for the treat-all strategy and 0 for treat-none. At a threshold of 0.50, 65.6% (63/96) of infants would be classified as high-risk, yielding the same sensitivity (0.871) but higher specificity (0.735), PPV (0.857), and NPV (0.758), and a net benefit of 0.469 versus 0.292 and 0 for the treat-all and treat-none strategies, respectively.

### 3.4. Model Interpretability

**Figure 5:**
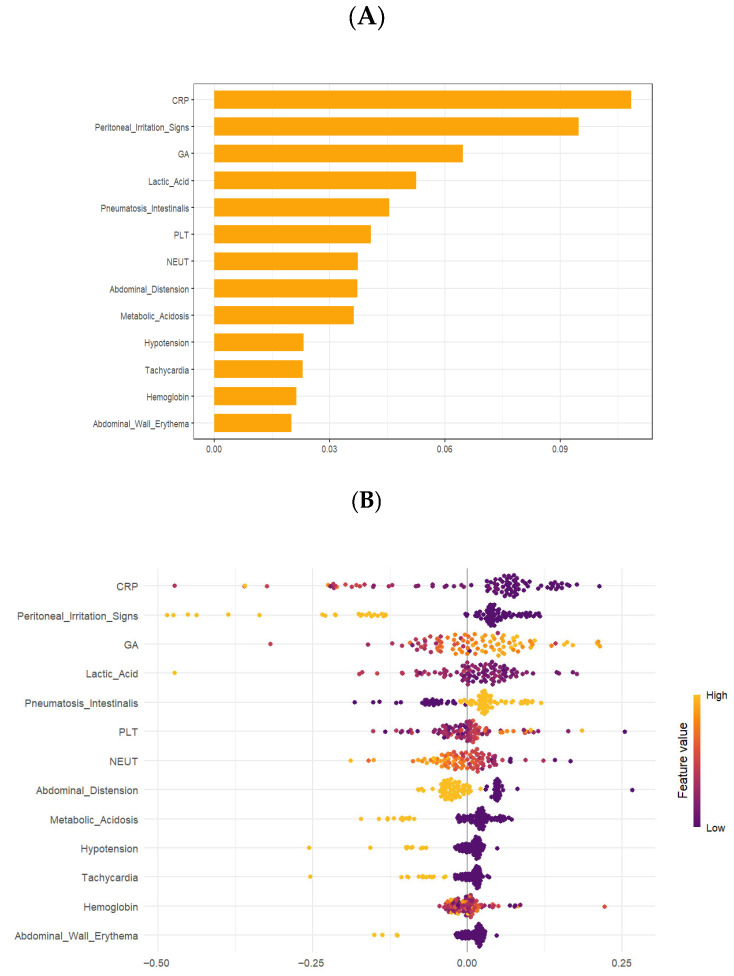
Global explanation by the SHapley Additive exPlanations (SHAP) values for the best-performing Neural Network model. (**A**) Global feature importance ranking (bar chart of the average SHAP value for each predictor); (**B**) beeswarm plots visualizing the distribution of SHAP values across all samples.

Model explainability was implemented through Shapley Additive exPlanations (SHAP) [16], which quantifies global and local feature importance under the optimal machine learning model. Figure 5 presents the global interpretability of the Neural Network model using SHAP analysis [16] based on the testing dataset. The SHAP summary plot and importance ranking collectively demonstrate the contribution and direction of each feature to the surgical decision prediction in neonates with NEC. As shown in the figure, CRP was identified as the most influential predictor, followed by peritoneal irritation signs, gestational age (GA), lactic acid, and pneumatosis intestinalis. Other important variables included platelet count (PLT), neutrophil percentage (NEUT), abdominal distension, and metabolic acidosis, while hypotension, tachycardia, hemoglobin, and abdominal wall erythema contributed less to the model output. The distribution of SHAP values further illustrates that elevated CRP levels and the presence of peritoneal irritation signs were strongly and positively associated with a higher probability of surgical intervention. For gestational age, the SHAP beeswarm plot suggested a nonlinear, approximately U-shaped relationship: extremely preterm infants tended to have a higher predicted probability of surgery, infants with moderately preterm gestational age showed a lower predicted risk, and a subset of late preterm infants with more severe clinical presentations also exhibited increased SHAP values. In contrast, higher platelet counts and hemoglobin levels tended to decrease the likelihood of surgery. These findings provide a transparent understanding of how each clinical feature influences the Neural Network’s prediction for NEC management decisions.

## 4. Discussion

In this study, we developed and validated machine learning (ML) models to predict the need for surgical intervention in preterm infants with necrotizing enterocolitis (NEC) using comprehensive clinical, laboratory, and imaging data. Our results demonstrate that ML approaches, particularly Neural Networks, Random Forest, and XGBoost, can achieve high predictive performance, providing objective tools to support clinical decision-making in this vulnerable population.

### 4.1. Key Findings

Among 320 preterm infants, 119 received operative management while 201 received non-operative care. Thirteen consensus features were identified as key predictors of surgical intervention, including gestational age, C-reactive protein (CRP), lactic acid, peritoneal irritation signs, pneumatosis intestinalis, hematologic parameters, and metabolic acidosis. Notably, CRP, peritoneal irritation signs, and gestational age emerged as the most influential factors in our Neural Network model based on SHAP analysis. Importantly, the effect of gestational age was nonlinear: extremely preterm infants and a subset of late preterm infants with more severe clinical presentations had higher predicted probabilities of surgery, whereas infants with moderately preterm gestational age showed relatively low risk, indicating an approximately U-shaped association with surgical intervention.

The Neural Network model achieved the highest overall classification metrics, with an accuracy of 0.875, sensitivity of 0.824, and specificity of 0.903. Random Forest achieved the highest ROC-AUC (0.922), while XGBoost demonstrated the highest PR-AUC (0.867), suggesting that different models may excel under different evaluation metrics. Overall, our findings highlight that integrating multidimensional data through ML can outperform traditional single-variable or logistic regression approaches, which often fail to capture nonlinear interactions among clinical predictors.

### 4.2. Clinical Implications

Our ML-based prediction framework offers several potential clinical benefits. First, it provides early identification of high-risk infants who may require surgery, thereby enabling timely intervention and potentially reducing morbidity and mortality. Second, the use of interpretable models with SHAP analysis allows clinicians to understand the contribution of individual features, facilitating trust and adoption in clinical settings. For instance, elevated CRP, metabolic acidosis, and radiological evidence of pneumatosis intestinalis could trigger closer monitoring and early surgical consultation. Third, such predictive models can help to standardize surgical decision-making, reducing reliance on subjective judgment and inter-clinician variability, which is particularly valuable in high-volume NICUs.

Building on these results, we propose a simple risk-stratification scheme to facilitate integration of the model into a routine clinical workflow. Based on the DCA findings, LightGBM-predicted probabilities can be mapped to three categories: low risk (<30%), intermediate risk (30–49%), and high risk (≥50%) of requiring surgical intervention. Infants in the low-risk group would continue to receive standard medical management and routine monitoring. Those in the intermediate-risk group would be flagged for early surgical consultation, closer hemodynamic and abdominal examination, and more frequent abdominal radiography or ultrasonography to detect disease progression. For infants with predicted risk ≥50%, the model output would prompt the clinical team to prioritize operative readiness—such as arranging operating-room availability, discussing possible laparotomy with families, and considering earlier surgical intervention if clinical findings remain concerning. In practice, the model could be embedded into the electronic medical record so that, if the 13 predictors are available at the time of NEC diagnosis, the predicted probability and risk category are automatically calculated and displayed using a color-coded alert. These proposed thresholds are intended as a starting point for prospective validation rather than definitive cutoffs, but they illustrate how the ML model can be incorporated into multidisciplinary NEC decision-making.

### 4.3. Comparison with Previous Studies

Studies indicate that 27% to 52% of NEC infants require surgery, including primary peritoneal drainage, exploratory laparotomy, or necrotic bowel resection [17,18,19]. Precise surgical timing critically influences mortality and complication rates, yet there exists a critical deficiency in objective predictive tools for optimal intervention timing [20], often resulting in consequential delays in clinical decision-making.

Extensive research has sought to identify predictors of surgical necrotizing enterocolitis (NEC) to optimize clinical management. Abdominal ultrasonography (AUS) has demonstrated value in the early detection of NEC, whereas bedside radiography (chest and abdominal) remains a cornerstone for surgical decision-making [21]. Biomarker-based predictive models incorporating platelet-to-lymphocyte ratio (PLR), white blood cell count (WBC), absolute neutrophil count (ANC), absolute lymphocyte count (ALC), neutrophil-to-lymphocyte ratio (NLR), C-reactive protein (CRP), and procalcitonin (PCT) have shown utility in distinguishing surgical from medical NEC cases [22]. Moreover, interleukins IL-6 and IL-8 have been proposed as potential markers for determining optimal surgical timing [23,24]. Previous multivariate analyses have identified low gestational age, early NEC onset, hemodynamically significant patent ductus arteriosus, hypobicarbonatemia, acidosis, and coagulopathy as independent risk factors for surgical intervention [25,26].

Compared with these earlier studies, our work offers several notable improvements. First, we systematically integrated 43 multidimensional features—including clinical, laboratory, imaging, and perinatal variables—and applied dual-method feature selection (LASSO and Boruta) to identify 13 consensus predictors. Second, we evaluated four machine learning (ML) algorithms—LightGBM, XGBoost, Random Forest, and Neural Network—and demonstrated the superior overall performance of the Neural Network in terms of accuracy, sensitivity, specificity, and balanced accuracy. Third, model interpretability was enhanced using SHapley Additive exPlanations (SHAP), enabling quantitative assessment of individual feature importance. CRP, abdominal distension, and peritoneal irritation signs emerged as high-impact predictors, highlighting an inflammation-dominant pathophysiological pattern.

Relative to previous NEC prediction models in preterm infants, our model exhibited substantially higher discriminative ability. For example, a Korean ML study predicting surgical NEC in very low-birth-weight infants reported an AUROC of 0.721 [27], whereas a Chinese study achieved an AUROC of 0.8 [28]. In contrast, our Neural Network model reached an AUROC of 0.916, indicating markedly improved predictive performance. Importantly, prior studies often included heterogeneous populations encompassing both preterm and term infants, potentially confounding predictive accuracy; by focusing exclusively on preterm neonates, our study addresses this critical gap.

Beyond NEC, ML and other artificial intelligence techniques have increasingly been applied in pediatric acute-care settings. For example, a recent study demonstrated that ML models and large language models can assist clinicians in predicting serious bacterial infections in febrile infants, thereby improving risk stratification in the emergency department [29]. Such work highlights a broader trend toward ML-assisted pediatric decision-support tools. Our NEC surgical prediction model fits within this landscape by extending ML methods to a high-risk neonatal population and focusing on a complex surgical decision, thus complementing existing applications in pediatric infectious diseases and sepsis.

### 4.4. Strengths

This study has several strengths. First, it is based on a relatively large, single-center cohort of preterm infants with NEC, ensuring consistent diagnostic criteria and clinical management protocols. Second, the integration of clinical, laboratory, and imaging data captures multidimensional aspects of disease severity. Third, the combination of multiple ML algorithms with hyperparameter optimization, cross-validation, and rigorous performance evaluation (ROC-AUC, PR-AUC, sensitivity, specificity, PPV, NPV) strengthens model reliability. Moreover, we complemented the primary LightGBM model with learning curves, bootstrap confidence intervals for ROC-AUC and PR-AUC, and a bootstrap-based feature-selection stability analysis, all of which consistently supported the robustness of the model despite the borderline events-per-variable ratio in the non-surgical group. Finally, the inclusion of SHAP analysis enhances interpretability, a key requirement for clinical adoption.

From a clinical workflow perspective, the proposed ML model is intended to be used as a point-of-care decision-support tool at the time of NEC diagnosis in the neonatal intensive care unit. The 13 predictors required by the model are routinely available from bedside clinical assessments, laboratory tests, and radiological imaging and can be automatically extracted from the electronic medical record or entered into a simple web-based or embedded calculator. The model would then output an individualized predicted probability of requiring surgical intervention, together with an easily interpretable risk category (e.g., low, intermediate, or high risk). High-risk infants could be prioritized for early pediatric surgical consultation, closer hemodynamic and abdominal monitoring, and timely repeat imaging, whereas lower-risk infants would continue to receive optimized medical management with ongoing surveillance.

### 4.5. Limitations

Several limitations should be acknowledged. First, the study is retrospective and single-center, which may limit generalizability to other populations or healthcare settings. Second, although missing data were imputed, potential biases due to incomplete records cannot be entirely excluded. Third, while ML models demonstrated high predictive performance, external validation in independent cohorts is required before clinical implementation. Fourth, the models focused on preterm infants; extension to term infants or broader populations requires further investigation. Finally, the ML framework does not replace clinical judgment but should be considered an adjunct to guide decision-making. Future studies should focus on multicenter prospective validation, integration with real-time electronic health records, and exploration of dynamic prediction models that incorporate longitudinal clinical data. Additionally, combining ML-based predictions with other biomarkers or imaging modalities may further enhance accuracy and clinical applicability.

### 4.6. Conclusions

In conclusion, we developed and internally validated four ML models that use 13 routinely available clinical, laboratory, and imaging variables to predict the need for surgical intervention in preterm infants with NEC. These models, particularly the Neural Network and Random Forest, showed good discrimination and calibration and identified clinically plausible risk factors such as CRP, peritoneal signs, gestational age, lactate, and pneumatosis intestinalis. By providing an individualized probability of surgery at the time of NEC diagnosis, the proposed tool has the potential to support early risk stratification, prompt multidisciplinary evaluation, and more timely operative decision-making in the NICU. However, our findings should be interpreted in light of several limitations, including the single-center retrospective design, the moderate sample size—especially of the non-surgical group—and the lack of external validation. Future studies should validate and recalibrate these models in multicenter cohorts, incorporate dynamic longitudinal data, and prospectively evaluate their impact on clinical decision-making and outcomes. If confirmed, ML-based decision-support systems could be integrated into electronic medical records to improve care for preterm infants with NEC.

## Figures and Tables

**Table 1:** Demographic and perinatal characteristics.

Variables	Total (*n* = 320)	Operative Group (*n* = 119)	Non-Operative Group (*n* = 201)	*p*
Demographics				
Age (days)	8.50 (2.00, 17.00)	10.00 (5.00, 17.00)	7.00 (1.00, 17.00)	0.02
Gestational Age (days)	235.00 (217.00, 248.00)	225.00 (210.00, 240.00)	239.00 (222.00, 250.00)	<0.001
Birth Weight (g)	1835.00 (1450.00, 2320.00)	1600.00 (1365.00, 2045.00)	2040.00 (1550.00, 2460.00)	<0.001
Onset Time of Necrotizing Enterocolitis (days)	10.00 (3.00, 18.75)	9.00 (4.00, 17.00)	11.00 (3.00, 19.00)	0.39
Male	168 (52.50)	61 (51.26)	107 (53.23)	0.73

**Table 2:** Clinical signs and comorbidities.

Variables	Total (*n* = 320)	Operative Group (*n* = 119)	Non-Operative Group (*n* = 201)	*p*
Perinatal Factors				
Premature Rupture of Membranes	101 (31.56)	32 (26.89)	69 (34.33)	0.17
Abnormal Amniotic Fluid	153 (47.81)	52 (43.70)	101 (50.25)	0.26
Abnormal Placental	153 (47.81)	52 (43.70)	101 (50.25)	0.26
Intrauterine Distress	34 (10.62)	13 (10.92)	21 (10.45)	0.89
Feeding Type				0.04
Formula Feeding	52 (16.56)	24 (20.51)	28 (14.21)	
Breast Feeding	179 (57.01)	72 (61.54)	107 (54.31)	
Mixed Feeding	51 (16.24)	11 (9.40)	40 (20.30)	
Fasting	32 (10.19)	10 (8.55)	22 (11.17)	
Symptoms and signs				
Abdominal Distension	212 (66.25)	113 (94.96)	99 (49.25)	<0.001
Gastric Retention	45 (14.06)	14 (11.76)	31 (15.42)	0.36
Decreased Bowel Sounds	217 (67.81)	102 (85.71)	115 (57.21)	<0.001
Tachycardia	43 (13.44)	31 (26.05)	12 (5.97)	<0.001
Hematochezia	142 (44.38)	43 (36.13)	99 (49.25)	0.02
Peritoneal Irritation Signs	66 (20.62)	55 (46.22)	11 (5.47)	<0.001
Abdominal Wall Erythema	17 (5.31)	16 (13.45)	1 (0.50)	<0.001
Abdominal Mass	5 (1.56)	3 (2.52)	2 (1.00)	0.55
Hypotension	24 (7.52)	19 (15.97)	5 (2.50)	<0.001
Underlying disease and treatment	152 (47.50)	52 (43.70)	100 (49.75)	0.30
Infectious Diseases	152 (47.50)	52 (43.70)	100 (49.75)	0.30
Patent Ductus Arteriosus	82 (25.87)	30 (25.64)	52 (26.00)	0.94
Antibiotics	152 (47.50)	52 (43.70)	100 (49.75)	0.13
Oral Probiotics	9 (2.90)	2 (1.69)	7 (3.65)	0.52
Intravenous Immunoglobulin	20 (6.25)	9 (7.56)	11 (5.47)	0.46
Diuretics	10 (3.14)	5 (4.27)	5 (2.49)	0.58
Vasoactive Drugs	28 (8.81)	8 (6.78)	20 (10.00)	0.33
Glucocorticoids	74 (23.20)	26 (22.03)	48 (23.88)	0.71
Caffeine	67 (21.07)	20 (17.09)	47 (23.38)	0.19
Pulmonary Surfactant Administration	66 (20.75)	26 (22.03)	40 (20.00)	0.67
Mechanical Ventilation	80 (25.00)	25 (21.01)	55 (27.36)	0.21
Peripherally Inserted Central Catheter	55 (17.24)	14 (11.86)	41 (20.40)	0.05

**Table 3:** Laboratory parameters.

Variables	Total (*n* = 320)	Operative Group (*n* = 119)	Non-Operative Group (*n* = 201)	*p*
Laboratory Parameters				
Lactic Acid (mmol/L)	2.20 (1.31, 3.67)	2.20 (1.50, 4.02)	2.10 (1.20, 3.30)	0.09
CRP (mg/L)	5.00 (2.83, 34.00)	43.00 (12.39, 91.00)	5.00 (1.00, 5.80)	<0.001
Neutrophil Percentage (%)	45.00 (31.07, 63.18)	57.60 (39.70, 68.75)	40.40 (28.45, 54.80)	<0.001
Platelet Count (×109/L)	247.50 (159.75, 351.50)	172.00 (107.00, 254.00)	279.00 (212.00, 390.00)	<0.001
Hemoglobin (g/L)	128.00 (111.00, 149.25)	118.00 (102.00, 131.50)	135.00 (116.00, 160.00)	<0.001
Metabolic Acidosis	45 (14.29)	32 (27.12)	13 (6.60)	<0.001
Hyponatremia	93 (29.34)	49 (41.18)	44 (22.22)	<0.001
Positive Blood Culture	15 (4.73)	9 (7.56)	6 (3.03)	0.07

**Table 4:** Imaging findings.

Variables	Total (*n* = 320)	Operative Group (*n* = 119)	Non-Operative Group (*n* = 201)	*p*
Imaging				
Pneumatosis Intestinalis	226 (70.62)	66 (55.46)	160 (79.60)	<0.001
Portal Venous Gas	90 (28.12)	23 (19.33)	67 (33.33)	0.007
Intestinal Wall Thickening	226 (70.62)	85 (71.43)	141 (70.15)	0.81
Intestinal Dilation	84 (26.33)	41 (34.45)	43 (21.50)	<0.001

Data are expressed as median (interquartile range) or *n* (%).

**Table 5:** Performance of the 4 ML models.

Metric	LightGBM	XGBoost	Random Forest	Neural Network
Accuracy	0.823	0.844	0.823	0.875
AUC	0.911 (0.851–0.961)	0.915 (0.852–0.962)	0.922 (0.870–0.967)	0.916 (0.861–0.967)
Sensitivity	0.735	0.765	0.735	0.824
Specificity	0.871	0.887	0.871	0.903
PR AUC	0.843	0.867	0.847	0.851
PPV	0.758	0.788	0.758	0.824
NPV	0.857	0.873	0.857	0.903
Balanced accuracy	0.803	0.826	0.803	0.863

Abbreviations: LightGBM, Light Gradient Boosting Machine; XGBoost, eXtreme Gradient Boosting Machine; AUC, area under the curve; PR AUC, area under the precision–recall curve; NPV, negative predictive value; PPV, positive predictive value.

## Data Availability

The original contributions presented in this study are included in the article. Further inquiries can be directed to the corresponding authors.

## Supplementary Materials

The following supporting information can be downloaded at: https://www.mdpi.com/article/10.3390/children13010021/s1, Figure S1: Lightgbm learning curve AUC. Figure S2: Lightgbm learning curve PR-AUC.
