# Supplementary figures and images for: Machine Learning-Based Prediction of Surgical Intervention in Preterm Infants with Necrotizing Enterocolitis: A Retrospective Cohort Study
Source: Children (Basel). 2025 Dec 22;13(1):21. doi: 10.3390/children13010021 (PMC12839886; doi:10.3390/children13010021)

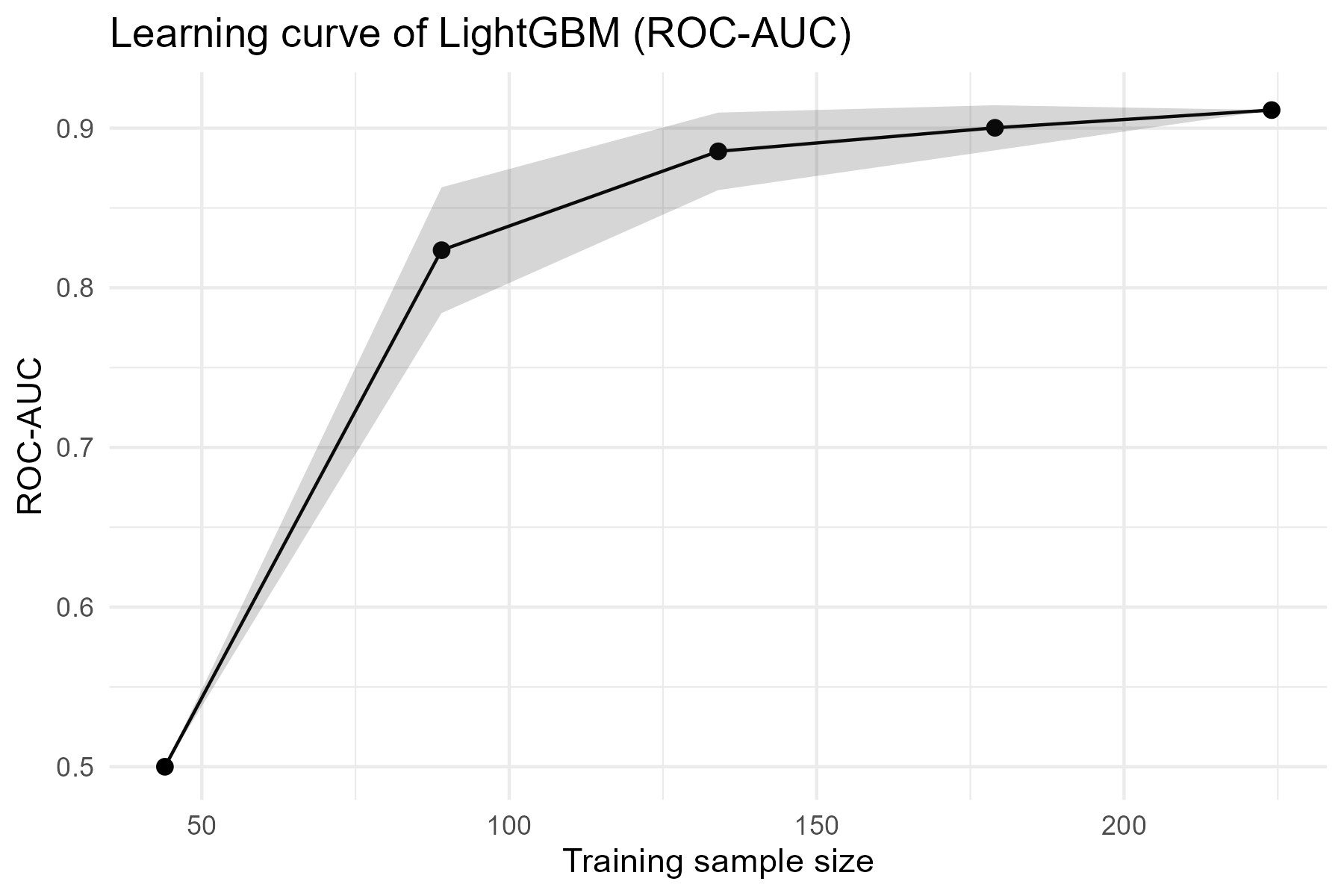

Supplement: Supplementary file 1 [file children-13-00021-s001.zip › Supplementary Figure S1. lightgbm_learning_curve_auc.png]

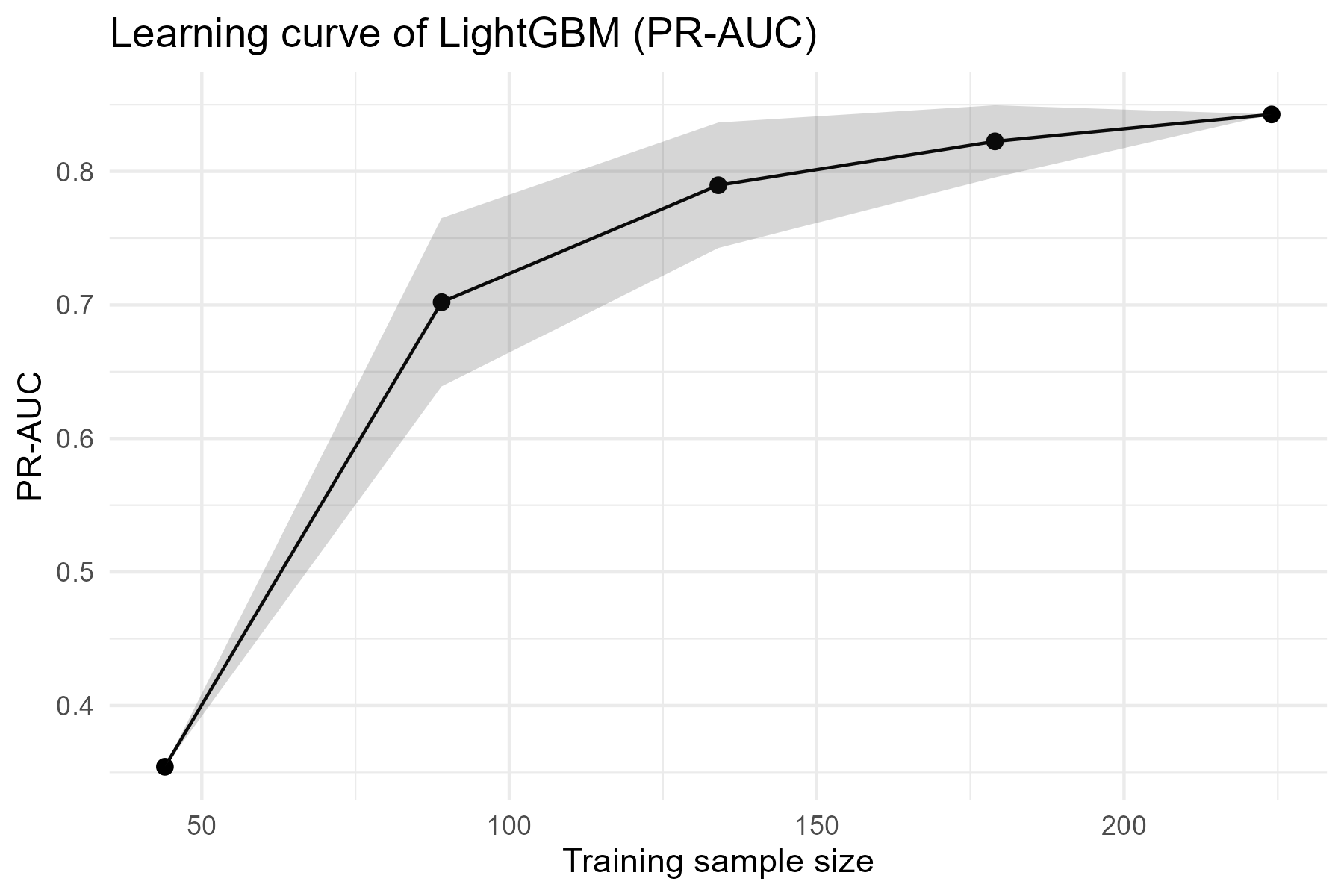

Supplement: Supplementary file 1 [file children-13-00021-s001.zip › Supplementary Figure S2. lightgbm_learning_curve_prauc.png]
